# Sirolimus for the Treatment of Juvenile Polyposis in Childhood

**DOI:** 10.14309/crj.0000000000000646

**Published:** 2021-08-25

**Authors:** Rafael Martín-Masot, Nerea Cardelo Autero, Pilar Ortiz Pérez, Encarnación Torcuato Rubio, Luis Vázquez Pedreño, Carmen Gallego Fernández, Javier Blasco-Alonso, Víctor Manuel Navas-López

**Affiliations:** 1 Pediatric Gastroenterology and Nutrition Unit, Hospital Regional Universitario de Málaga, Málaga, Spain; 2 Endoscopy Department, Hospital Regional Universitario de Málaga, Málaga, Spain; 3 Pharmacy Department, Hospital Regional Universitario de Málaga, Málaga, Spain

## Abstract

Juvenile polyposis syndrome (JPS) is a rare disease with an autosomal dominant inheritance pattern characterized by the development of multiple hamartomatous polyps in the gastrointestinal tract. The most frequent signs and symptoms are recurrent abdominal pain, rectal bleeding, anemia, and iron deficiency. The treatment of JPS is symptomatic, requiring serial endoscopic polypectomies or intestinal resections in the most severe cases. We describe the clinical case of a patient with JPS with a childhood juvenile polyposis phenotype because of a mutation on the SMAD4 gene, who received treatment with sirolimus successfully.

## INTRODUCTION

Juvenile polyposis syndrome (JPS) is an autosomal dominant disease with an estimated incidence of 1 per 100,000 inhabitants, characterized by the development of multiple hamartomatous polyps in the gastrointestinal tract.^1,2^ The clinical presentation is variable; the most frequent signs and symptoms are recurrent abdominal pain, rectal bleeding, anemia, and iron deficiency. JPS is diagnosed according to genetic and clinical features in the absence of extraintestinal characteristics compatible with PTEN hamartoma tumor syndrome. JPS is associated with a higher risk of suffering from gastrointestinal malignancies.^3,4^ Currently, the treatment of JPS is symptomatic, requiring serial endoscopic polypectomies or intestinal resections in the most severe cases, as well as treating the complications derived from the disease.^5^ Until now, no pharmacological treatment has been approved to treat this disease. To the best of our knowledge, we present the first case in the literature of a patient with JPS and a mutation in the SMAD4 gene with the resolution of symptoms and colonic polyps after receiving treatment with sirolimus.

## CASE REPORT

**Figure 1.**
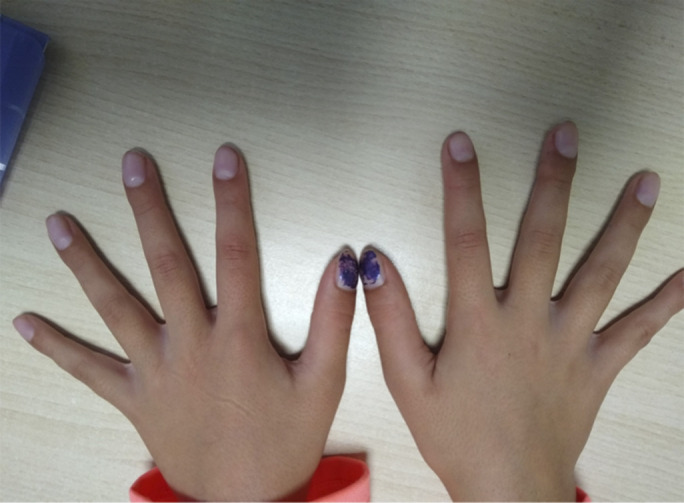
Image of the patient's hands showing clubbing.

**Figure 2.**
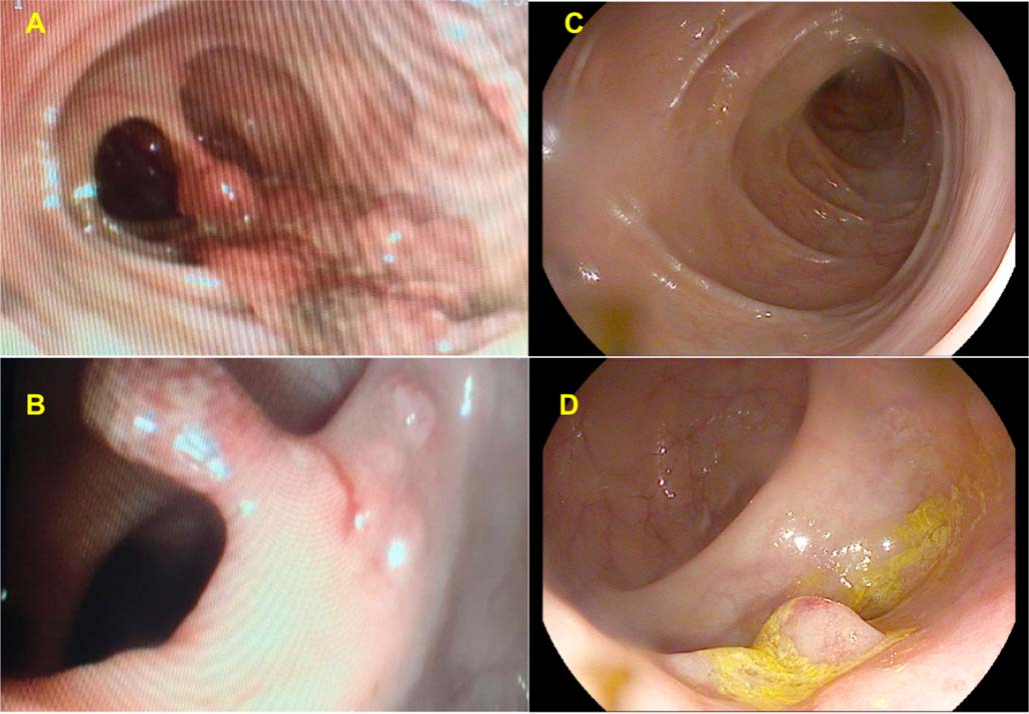
(A and B) Images corresponding to the colonoscopy performed at the diagnosis of juvenile polyposis syndrome. Numerous large pedunculated polyps are evident, some of them even establishing mucosal bridges. (C and D) Images corresponding to the colonoscopy performed 9 months after starting treatment with sirolimus, in which only small sessile polyps were evidenced.

An 8-year-old girl was referred to a pediatric gastroenterology clinic for chronic microcytic anemia of 2 years of evolution, bloody diarrhea (4–5 bowel movements a day), and growth retardation. At diagnosis, normal physical examination except for mucocutaneous paleness and the presence of clubbing on the hands and feet was observed (Figure 1). Blood and stool tests showed anemia (Hb 8.9 g/dL), iron deficiency (11 μg/dL), and elevated fecal calprotectin (903 μg/g). The endoscopy showed multiple polyps from the rectum to the cecum (>20–30 polyps with a maximum size of 2–3 cm) (Figure 2) and histology compatible with JPS. The genetic study revealed a mutation in the SMAD4 gene (c.1157G> A in exon 10).

After diagnosis, the patient received intravenous iron and serial endoscopic polypectomies (every 4–6 months). Seven endoscopies were performed, resecting between 12 and 20 polyps in each of them, leaving >30 remaining polyps in the colon after the last session. At this point, after 4 years of evolution, given the undesirable effects of performing serial endoscopies, it was decided to start treatment with sirolimus. An initial dose of 0.8 mg/m^2^/d was used. However, this dose had to be increased throughout the first month to 1.5 mg/m^2^/d to reach therapeutic levels (6–10 ng/mL), according to as described by other authors.^6–8^ Since treatment, an improvement in laboratory parameters was observed, and the administration of intravenous iron was suspended. After 12 months of treatment with sirolimus, laboratory test values were within normal ranges (Hb 14.1 g/dL, iron 43 μg/dL, and fecal calprotectin 22.7 μg/g).

Nine months later from the start of treatment with sirolimus, a control colonoscopy was performed to assess the response to treatment, showing the disappearance of almost all the polypoid lesions, as well as a drastic reduction in the size of the remnants. From the rectum to the cecum, a maximum of 12 polyps were identified, all of them 2–5 mm at most (Figure 2) that were not removed. Clinical improvement was also observed. To date, after 1 year of follow-up, the patient has not presented any of the most frequent side effects described by the use of this immunomodulator (lipid disorders, cytopenia, hepatotoxicity, nephrotoxicity, increased incidence of infections, interstitial lung disease, or thrombotic microangiopathy).

## DISCUSSION

**Figure 3.**
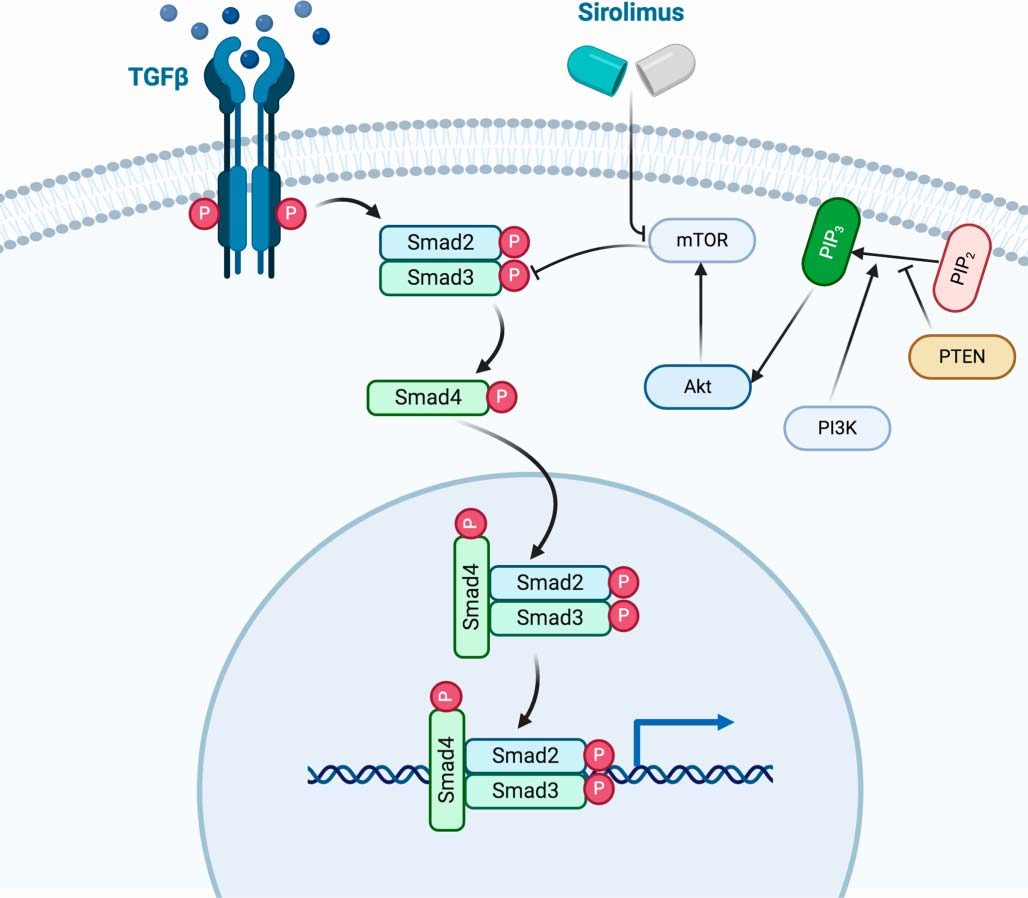
Schematic diagram of the hypothetical mechanism of action of sirolimus. Figure created with BioRender.com.

Patients with JPS present mutations in the SMAD4 (18q21.1) or BMPR1A (10q23.2) genes in up to 40%–60% of cases, being de novo in approximately 25%.^9,10^ Furthermore, mutations at the level of the tumor suppressor gene PTEN (telomeric to BMPR1A) and the ENG gene in young patients with JPS in whom no mutation was found at the level of SMAD4 or BMPR1A have been identified.^11-13^ In our patient, a mutation at SMAD4 gene (c.1157G> A in exon 10), described for the first time in the literature in 2004 was identified. Loss of heterozygosity of SMAD4 in the epithelium initiates polyp growth, suggesting that SMAD4 acts as a classic tumor suppressor protein in JPS polyps.^14,^^15^ In this way, mutations in this gene imply a lower production of the transforming growth factor-beta protein, cytosine involved in inhibiting cell growth and proliferation.^16^ On the other hand, the loss of function at the level of PTEN, another of the genes that is related to this disease, is associated with greater activation of the protein kinase B pathway (mammalian target of rapamycin [mTOR]), which is also involved in cell proliferation.^7^ Although these are different mutations, both pathways are related because m-TOR interacts with the transforming growth factor-beta pathway, blocking it and, therefore, further promoting the inhibition of cell apoptosis.^16^ This justifies the use of sirolimus, an mTOR inhibitor drug, in patients with JPS with mutations in the SMAD4 gene, not only if there are mutations in the PTEN (Figure 3).

The significant impact on the quality of life and the absence of disease-modifying treatment justifies the search for new mechanisms to treat these diseases effectively and safely. Studies have recently been published that have shown, both in animal models and in humans, the effectiveness of sirolimus to inhibit the formation of gastrointestinal polyps and increase the time of progression to dysplasia in patients with intestinal polyposis syndromes, including JPS.^6–8,17^ Table 1 details the characteristics of the main studies available in the literature to date. This drug is an inhibitor of mTOR, a molecule directly related to an increase in cell proliferation and, therefore, responsible for polyps in patients with JPS.^18^

**Table 1.** Studies in which sirolimus has been used for the treatment of polyposis syndromes

	Hardiman et al.^17^	Yuksekkaya et al.^8^	Busoni et al.^7^	Quaranta et al.^6^
Year	2014	2016	2019	2019
Disease	FAP	FAP	JPS, juvenile polyposis of infancy phenotype	JPS, juvenile polyposis of infancy phenotype
Type of study	Case-control	Case series	Clinical case	Clinical case
Patients (n) Age	12 (6 cases and 6 controls)	2 (13 and 14 yr old)	1 (20 mo old)	1 (6 yr old)
Gene	APC	APC	PTEN BMPR1A	PTEN BMPR1A
Sirolimus dose	3 mg/kg/d	0.05–0.1 mg/kg	0.8 mg/m^2^/d	—
Target sirolimus levels (ng/mL)	—	3–6	6–8	5
Study duration	2 wk	40 mo	27 mo	4 yr
Side effects	No	No	No	No
Results	• Improved survival, less formation of colonic polyps, less dysplasia, and later onset in the group treated with sirolimus. • Treatment with sirolimus alters mTOR signaling, a marker of cell proliferation and differentiation.	• Reduction of the size of colonic polyps, as well as the severity of dysplasia.	• Reduction in the number and size of polyps. • The patient did not require new transfusions of red blood cells or albumin. • Growth improvement • Better QOL (patient and the family).	• Decrease in the number and size of polyps. • Anthropometric and laboratory values (hemoglobin and albumin) improvement
Comments	• Study conducted in mice. • Formation of polyps induced with tamoxifen.	—	—	—

FAP, familial adenomatous polyposis; JPS, juvenile polyposis syndrome; mTOR, mammalian target of rapamycin; QOL, quality of life.

Sirolimus is a drug with an adequate long-term safety profile, widely used in other pathologies, such as lymphangioleiomyomatosis or solid organ transplantation.^19^ Blood tests and fecal calprotectin are performed every 4 months to detect the most frequent side effects of sitolimus and endoscopic controls annually to confirm the nonappearance of new polyps and as part of the colorectal cancer surveillance program.^20^ Because of the significant improvement produced both physically and in terms of quality of life, we currently consider a risk-benefit balance superior to not using the medication, considering long-term maintenance of it.

The growing evidence for the use of sirolimus could make it a valid alternative to endoscopic treatment of patients with JPS, potentially avoiding colectomy. Because of its serious consequences, the use of sirolimus opens up a new treatment pathway unknown until now, although it is advisable to be rigorous in the follow-up of these patients. It will be necessary to establish its long-term efficacy because of the probability of developing resistance and multicenter studies evaluating its safety and effectiveness in larger patient cohorts.

## DISCLOSURES

Author contributions: All authors contributed equally to this manuscript. VM Navas-López is the article guarantor.

Financial disclosure: None to report.

Informed consent was obtained for this case report
